# Circumvention of multi-drug resistance of cancer cells by Chinese herbal medicines

**DOI:** 10.1186/1749-8546-5-26

**Published:** 2010-07-25

**Authors:** Stella Chai, Kenneth KW To, Ge Lin

**Affiliations:** 1 School of Biomedical Sciences, Faculty of Medicine, The Chinese University of Hong Kong, Shatin, Hong Kong SAR, China; 2 School of Pharmacy, Faculty of Medicine, The Chinese University of Hong Kong, Shatin, Hong Kong SAR, China

## Abstract

Multi-drug resistance (MDR) of cancer cells severely limits therapeutic outcomes. A proposed mechanism for MDR involves the efflux of anti-cancer drugs from cancer cells, primarily mediated by ATP-binding cassette (ABC) membrane transporters including P-glycoprotein. This article reviews the recent progress of using active ingredients, extracts and formulae from Chinese medicine (CM) in circumventing ABC transporters-mediated MDR. Among the ABC transporters, Pgp is the most extensively studied for its role in MDR reversal effects. While other MDR reversal mechanisms remain unclear, Pgp inhibition is a criterion for further mechanistic study. More mechanistic studies are needed to fully establish the pharmacological effects of potential MDR reversing agents.

## Review

### Multi-drug resistance (MDR)

**Figure 1:**
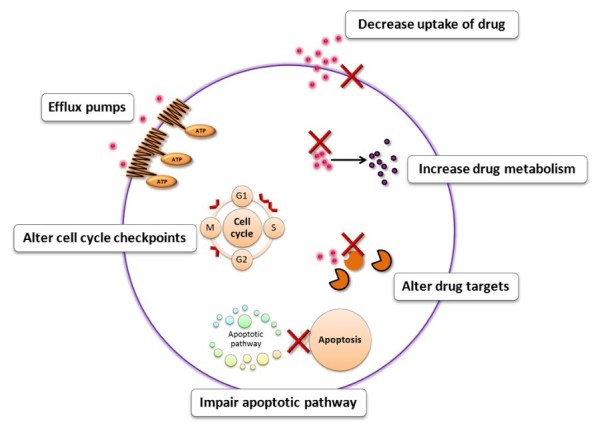
**Mechanisms of MDR towards cancer chemotherapeutic drugs**. Cancer cells can develop resistance to multiple drugs by various mechanisms as depicted. Mechanisms include (a) decreased uptake of drug, (b) reduced intracellular drug concentration by efflux pumps, (c) altered cell cycle checkpoints, (d) altered drug targets, (e) increased metabolism of drug and (f) induced emergency response genes to impair apoptotic pathway.

**Figure 2:**
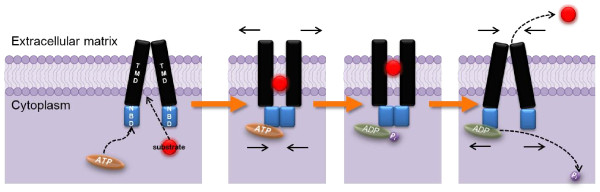
**Proposed drug efflux mechanism for ABC transporters**. Substrate and ATP bind to ATP transporters. After ATP hydrolysis, the substrate is effluxed out of the cell. Phosphate group is released and the substrate is then excreted to extracellular matrix.

Multi-drug resistance (MDR) in cancer chemotherapy refers to the ability of cancer cells to survive from treatment of a wide range of drugs [1]. In addition to the MDR induced by drugs in early exposure, the MDR cancer cells may subsequently develop cross-resistance to several unexposed and structurally unrelated chemotherapeutic agents [2]. Mechanisms of MDR include decreased uptake of drugs, alterations in cellular pathways and increased active efflux of drugs [3-5] (Figure 1). Overexpression of ATP-binding cassette (ABC) transporters is one of the most common mechanisms. ABC transporters are large membrane-bound proteins consisting of two nucleotide-binding domains (NBDs) and two transmembrane domains (TMDs) which mediate the active transport of substrate drugs out of the cell (Figure 2). Overexpression of the three major ABC transporters, i.e. P-glycoprotein (Pgp), multidrug-resistance-associated protein 1 (MRP1) and breast cancer resistance protein (BCRP/ABCG2) is frequently observed in cancer cell lines selected with chemotherapeutic drugs [6] and critical to clinical drug resistance [7].

#### P-glycoprotein (Pgp)

P-glycoprotein (Pgp) [8], which is also referred to as ABCB1 and MDR1, is the most studied ABC transporter. Pgp transports a wide range of chemotherapeutic agents including the anthracyclines, vincas, taxanes, etoposide and mitoxantrone [6]. Pgp is expressed in various tissues in the body. Remarkably high expression can be found in endothelial cells of capillary blood vessels in the brain as well as other organs including intestines, testes and skin [9,10]. Pgp expression is often detected in renal carcinoma, colon carcinoma, adrenal carcinoma and teratocarcinoma [9]. Substrate drugs can bind to Pgp through multiple binding sites, thereby allowing flexibility in the mechanism of transport [11,12].

#### Multidrug-resistance-associated protein 1 (MRP1)

The second major MDR transporter, multidrug-resistance-associated protein (MRP), was first discovered in a doxorubicin-selected lung cancer cell line [13]. A member of the ABCC subfamily, MRP1 is encoded by the *ABCC1 *gene [14]. Physiologically, MRP1 tends to pump drugs into the body, rather than excreting them into the bile, urine or gut [15,16]. MRP1 was highly expressed in skeletal muscles [17]. Overexpression of MRP1 is in cancer types such as lung, colon and various forms of leukaemia [18].

#### Breast cancer resistance protein (BCRP/ABCG2)

Recently, ABCG2 was identified in cancer cell lines selected with mitoxantrone that do not express Pgp and MRP1. As ABCG2 was simultaneously discovered by several research groups, it was also named BCRP, ABCP and MXR [19-21]. ABCG2 is expressed in a range of tissues, most abundantly in the liver and intestinal epithelia [22,23]. ABCG2 is localized in the apical region in cells [24] and transports many cytotoxic drugs, detoxified metabolites, toxins and carcinogens [25].

### Chinese medicine and MDR

How to tackle the MDR cells in chemotherapy is a pressing issue in cancer treatments. Verapamil was the first known Pgp inhibitor to increase the intracellular concentration of anticancer agents in MDR cells by binding to Pgp and inhibiting the Pgp-mediated efflux [26]. It was believed that anticancer drug resistance could be reversed by drug efflux inhibition. Researchers developed and tested a range of Pgp inhibitors to improve the pharmacological effects of chemotherapy in cancer patients [27-29]. However, none of these Pgp inhibitors was further developed for clinical use. Many researchers are looking into Chinese medicine (CM) for potential MDR reversing agents.

This article reviews some of the recent findings on the circumvention of ABC transporters-mediated MDR by various ingredients and extracts of CM and their formulae based on whether the MDR reversal involved Pgp alteration.

### MDR reversal involving Pgp inhibition

#### Active ingredients - alkaloids

Tetrandrine, a calcium channel blocker, is a bisbenzylisoquinoline alkaloid isolated from the root of *Stephania tetrandra *(Fenfangji) [30]. Tetrandrine reversed MDR *in vitro *and modulated Pgp-mediated drug efflux [30-33]. A combination of tetrandrine with doxorubicin or vincristine *in vitro *demonstrated synergistic anticancer effects [34]. Tetrandrine reduced Pgp expression [35]. In mice bearing resistant MCF-1/DOX cells, tetrandrine potentiated the antitumor activities of doxorubicin without significantly increasing toxicity [36]. A synthetic halogenated form of tetrandrine increased vinblastine accumulation in a dose-dependent manner in resistant P388/DOX cell line and prolonged the life-span of tumour-bearing mice up to 25% without any side effects [37]. In a recent clinical trial, a combination of tetrandrine with daunorubicin, etoposide and cytarabine demonstrated antileukaemic effects in 38 patients with acute myeloid leukaemia [38].

Matrine is a quinolizidine alkaloid from *Sophora alopecuroides *(Kudouzi). In resistant K562/DOX cell line, matrine (up to 50 μg/mL, non-toxic) increased the intracellular accumulation of doxorubicin and induced its apoptotic effects [39]. Matrine enhanced the cytotoxicity of vincristine in resistant K562/VCR cell line [40]. It was proposed that matrine circumvented MDR by reducing Pgp expression [35].

Tetramethylpyrazine is an active alkaloid from *Ligusticum chuangxiong *(Chuanxiong) and a calcium channel blocker [41]. In Pgp-overexpressing resistant HL-60/VCR cell line, tetramethylpyrazine significantly reversed MDR towards various drugs such as vincristine, duanorubicin and doxorubicin [42]. Tetramethylpyrazine reduced drug efflux (up to 50%) in Pgp-overexpressing resistant MCF-7/DOX cell line [43]. When used together with β-elemene, tetramethylpyrazine exhibited stronger MDR reversal effects in resistant K562/DOX cell line [44]. Tetramethylpyrazine decreased Pgp expression in resistant HepG2/DOX cell line [45]. However, the reduction in Pgp expression was not universally observed. For instance, Pgp level was not altered despite MDR reversal in tetramethylpyrazine-treated K562/DOX cells [46].

Peimine (also known as verticine) is a cevanine type isosteroidal alkaloid from the bulbs of *Fritillaria thunbergii *(Zhebeimu) and other *Fritillaria *species [47,48]. In resistant K562/DOX and HL-60/DOX cell lines, peimine increased intracellular concentration of daunorubicin and reversed MDR probably through inhibition of Pgp expression [49].

Berbamine is a calcium channel blocker from *Mahonia fortunei *(Shidagonglao). In K562/DOX cell line, berbamine inhibited cell growth by inducing apoptosis in a dose-dependent manner and reduced Pgp expression thereby increasing the intracellular concentration of rhodamine-123 and doxorubicin [50,51]. In MCF-7/DOX cell line, O-(4-ethoxyl-butyl)-berbamine, a derivative of berbamine, reversed MDR by enhancing G2/M arrest and increasing the intracellular accumulation of doxorubicin [52].

#### Active ingredients - saponins

Ginsenosides are the major active components from *Panax ginseng *(Renshen). Ginsenosides are mainly triterpenoid dammarane derivatives. Several ginsenosides, namely Rg_1_, Rg_3_, Re, Rc and Rd inhibited drug efflux [53]. A combination of purified saponins containing Rb_1_, Rb_2_, Rc, Rd, Re and Rg_1 _reversed MDR whereas individual ginsenosides did not produce any effect [54]. Ginsenosides reversed MDR of several chemotherapeutic drugs such as homoharringtonine, cytarabine, doxorubicin and etoposide in K562/VCR and in a dose-dependent manner in K562/DOX [55]. Pgp expression decreased but bcl-2 expression remained the same [56]. Rb_1 _reversed MDR of harringtonolide and vincristine in K562/HHT and HL60/VCR cell lines respectively [57,58].

*Panax notoginseng *(Sanqi) total saponins reversed MDR of doxorubicin in MCF-7/DOX and K562/VCR cell lines. The mechanism may be related to the decrease of Pgp expression [59,60].

#### Active ingredients - flavonoids

Quercetin is one of the most widely distributed flavonoids in natural products including Chinese medicinal herbs such as *Sophora japonica *(Huai). Quercetin inhibited the binding of heat shock factor at the *MDR1 *promoter, thereby decreasing *MDR1 *transcription and reducing Pgp expression [61]. Quercetin also inhibited the overexpression of Pgp mediated by arsenite [62]. In HL-60/DOX and K562/DOX cell lines, quercetin enhanced the anticancer sensitivity to daunorubicin and decreased Pgp expression [63,64]. MDR reversal effect of quercetin was probably mediated by its action on mitochondrial membrane potential and the induction of apoptosis. Furthermore, quercetin derivatives rather than quercetin itself reversed MDR [65]. Quercetin increased the sensitivity of Pgp-overexpressing KBV1 cell line towards vinblastine and paclitaxel in a dose-dependent manner. Among many active flavonoids, quercetin was less potent than kaempferol but more effective than genistein and daidzein in reversing MDR. Genistein and daidzein had no effect on Pgp expression [66]. Although quercetin may be a potential MDR reversing agent, lethal drug-drug interaction between quercetin and digoxin has been reported. Quercetin (40 mg/kg) elevated the peak blood concentration of digoxin and caused sudden death of tested animals [67].

Curcumin, the major component in *Curcuma longa *(Jianghuang), inhibited the transport activity of all three major ABC transporters, i.e. Pgp, MRP1 and ABCG2 [68]. Curcumin reversed MDR of doxorubicin or daunorubicin in K562/DOX cell line and decreased Pgp expression in a time-dependent manner [69]. Curcumin enhanced the sensitivity to vincristine by the inhibition of Pgp in SGC7901/VCR cell line [70]. Moreover, curcumin was useful in reversing MDR associated with a decrease in bcl-2 and survivin expression but an increase in caspase-3 expression in COC1/DDP cell line [71]. The cytotoxicity of vincristine and paclitaxel were also partially restored by curcumin in resistant KBV20C cell line [72]. Curcumin derivatives reversed MDR by inhibiting Pgp efflux [72]. A chlorine substituent at the *meta-*or *para*-position on benzamide improved MDR reversal [72]. Bisdemethoxycurcumin modified from curcumin resulted in greater inhibition of Pgp expression [73]. Tetrahydrocurcumin, the major metabolite of curcumin, inhibited all three major ABC transporters [74]. Curcumin induced atypical and caspase-independent cell death in MDR cells [75]. In leukaemic cells collected from 78 childhood leukaemia patients, curcumin reduced Pgp expression [76]. A specialized nanoemulsion of curcumin is better than conventional solution form drugs in enhancing the efficiency of drug delivery into the cells, down-regulating Pgp expression, inhibiting the NFκB pathway and promoting apoptotic response [68,77].

#### Active ingredients - others

Schizandrins, the active constituents of *Schisandra chinensis *(Wuweizi), were investigated for their MDR reversal effects. Schizandrin A was the most potent in reversing MDR by enhancing apoptosis and down-regulating Pgp and total protein kinase C expression. The crude extract of *Schisandra chinensis *reversed the resistance against vincristine *in vivo *[78]. Deoxyschizandrin and γ-schizandrin, among the nine dibenzo[a,c]cyclooctadiene lignans examined, enhanced intracellular drug concentration and induced cell cycle arrest at the G2/M phase when combined with sub-toxic dosages of doxorubicin [79]. Gomisin A, on the other hand, altered Pgp-substrate interaction by binding to Pgp simultaneously with substrates [80].

#### Formulae - injections

'Shengmai Injection', consisting of *Panax ginseng *and *Ophiopogon japonicus *(Maidong), down-regulated Pgp expression in peripheral blood lymphocyte membrane. When used together with oxaliplatin, 5-fluorouracil or folinic acid, the injection prolonged the survival rate of colon cancer patients [81]. The injection also enhanced the efficacy of tamoxifen and nifedipine in combination therapy [82].

#### Formulae - powders

'Shenghe Powder', consisting of *Panax ginseng*, *Scorophularia ningpoensis *(Xuanshen) and *Atractylodes macrocephala *(Baizhu), increased the intracellular concentration of vincristine in resistant SGC-7901/VCR cell line, possibly due to the induction of apoptosis and down-regulation of Pgp and bcl-2 expression [83].

'Modified Sanwubai Powder', consisting of herbs such as *Croton tiglium *(Badou), *Platycodon grandiflorum *(Jiegeng) and *Fritillaria thunbergii*, induced apoptosis in SGC-7901 cell line and down-regulated the gene expressions of p53, bcl-2, rasP21CD44 and Pgp [84].

#### Formulae - others

Three herbal extracts used to treat diseases other than cancer, namely Ams-11, Fw-13 and Tul-17, greatly enhanced the efficacy of vincristine both *in vitro *and *in vivo *and reversed MDR in a dose-dependent manner. Tul-17 inhibited Pgp expression [85].

Oil emulsion from *Brucea javanica *(Yadanzi) reversed MDR when used together with other chemotherapeutic drugs such as vincristine, doxorubicin, cisplatin, mitomycin C, 5-fluorouracil or etoposide, probably due to down-regulation of Pgp expression or inhibition of TOPO II or both [86,87].

'Sangeng Mixture Decoction', consisting of *Reynoutria japonica *(Huzhang), *Actinidia arguta *(Mihouligen) and *Geum aleppicum *(Shuiyangmeigen), reversed MDR of doxorubicin via down-regulation of Pgp expression [88].

FFTLG, a formula containing *Actinidia arguta*, reversed MDR in K562/DOX cell line by increasing the intracellular doxorubicin concentration [89].

R1, consisting of *Ligusticum chuanxiong*, *Curcuma longa *and *Millettia dielsiana *(Jixueteng), enhanced the anticancer activities of doxorubicin in MCF-7/DOX via down-regulation of Pgp expression [90,91].

When tested with doxorubicin, 5-fluorouracil and epirubicin in HepG2/DOX cell line, Ganai-1, a commercial product, reversed MDR via down-regulation of Pgp expression [92]. Another commercial product, Tianfoshen, decreased Pgp expression in K562/DOX cell line and reversed MDR of doxorubicin [93]. An umbilical plaster used with 5-fluorouracil, mitomycin C or cisplatin reversed MDR via down-regulation of Pgp expression [94].

### MDR reversal not related to Pgp alteration

#### Active ingredients - alkaloids

Dauricine is a bisbenzylisoquinoline alkaloid isolated from the root of *Menispermum dauricum *(Bianfuge) as a calcium channel blocker. Dauricine reversed vincristine resistance in MCF-7/DOX cell line [95]. However, dauricine did not alter Pgp expression in K562/DOX cell line [96]. Moreover, dauricine enhanced the cytotoxic effects of doxorubicin in HL60/HAR cell line without increasing the intracellular concentration of doxorubicin or inhibiting Pgp overexpression [97].

Daurisoline, a structural analogue of dauricine, is also a calcium channel blocker isolated from the root of *Menispermum dauricum*. Both dauricine and daurisoline sensitized MCF-7/DOX cell line towards doxorubicin and vincristine in a dose-dependent manner [98]. The MDR reversal effects of dauricine and daurisoline are comparable to those of verapamil and both alkaloids do not cause cardiovascular adverse effect [99].

#### Active ingredients - saponins

20(S)-Ginsenoside Rg_3_, one of the active ginsenosides from *Panax ginseng*, restored the sensitivity of resistant KBV20 cell line to various anticancer drugs, including vincristine, doxorubicin, etoposide and colchicine in a time-and dose-dependent manner. This ginsenoside competitively inhibited the binding of substrate drugs to Pgp and its binding affinity to Pgp was remarkably higher than that of verapamil. In contrast to the dose-dependent effects *in vitro*, 20(S)-ginsenoside Rg_3 _increased animal life span in an *in vivo *MDR model in a dose-independent manner [53].

#### Active ingredients - flavonoids

Paeonol is a weak calcium channel blocker isolated from the root of *Paeonia suffruticosa *(Mudan). In K562/DOX cell line, paeonol showed positive MDR reversal effect towards doxorubicin, daunorubicin, vincristine and vinblastine without modulating Pgp expression [100]. In parental K562 cells, paeonol induced apoptosis in a time-and dose-dependent manner [101].

#### Formulae - injections

'KLT Injection' consisting of the extract of *Coix lacryma-jobi *(Yiyi) enhanced the anticancer activities of paclitaxel and docetaxel and reversed MDR in a dose-dependent manner [102].

#### Formulae - others

'Siwu Mixture', consisting of *Paeonia lactiflora *(Shaoyao), *Rehmannia glutinosa *(Dihuang*)*, *Angelica sinensis *(Danggui) and *Ligusticum chuanxiong*, reversed doxorubicin MDR without altering Pgp expression in K562/DOX cell line [103].

### Other mechanisms

#### Active ingredients

Pseudolaric acid B, a major active component of *Pseudolarix kaempferi *(Jinqiansong), reversed MDR *in vitro *and *in vivo *and induced apoptosis via cell cycle arrest at G2/M phase. In either resistant cell line or nude mice model, pseudolaric acid B circumvented MDR associated with Pgp overexpression [104].

Salvinal, isolated from *Salvia miltiorrhizae *(Danshen), induced apoptosis and inhibited tubulin polymerization in various cancer cell lines, including the Pgp and MRP-overexpressing MDR cells [105].

A study on 22 compounds from CM herbs found that homoharringtonine, artesunate and bufalin from *Cephalotaxus hainanensis *(Hainancufei), *Artemisia annua *(Qinghao) and *Bufo marinus, B. viridis *(Chanchu) respectively exhibited active MDR modulation [106]. Moreover, other compounds such as jatrorrhizine, indirubin, rhynchophylline [107], arsenic trioxide [108,109], psoralen [110,111], oridonin [99,112], β-elemene [113,114] also showed MDR reversal effects.

#### Extracts

Nine out of 20 extracts of *Ganoderma *species including *G. lucidum *(Lingzhi) were cytotoxic and induced apoptosis similar to etoposide and doxorubicin which are commonly used in chemotherapy. In etoposide-selected resistant cell line H69, *G. lucidum *extract increased the sensitivity to etoposide and doxorubicin significantly, possibly due to increased intracellular DNA fragmentation and caspases 3 and 9 activities [115]. Moreover, extracts of *Glycyrrhiza glabra *(Gancao), *Hedyotis diffusa *(Baihuasheshecao) and *Rheum palmatum *(Dahuang) reversed MDR by increasing the intracellular concentration of daunorubicin in SGC7901/VCR cell line [116].

#### Formulae

'Ganli Injection', consisting of matrine and tetramethylpyazine hydrochloride, reversed MDR by increasing the sensitivity of 5-fluorouracil and the intracellular concentration of doxorubicin in BEL-7402/5-FU cell line [117].

'Bushen Huayu Jiedu Formula', consisting of *Cinnamomum cassia *(Rougui)*, Psoralea corylifolia *(Buguzhi) and *Rheum palmatum*, was tested in A549/DDP cell line and S180 tumour-bearing mice. *In vitro*, the formula significantly increased the intracellular concentration of cisplatin at high doses and inhibited the activity of calcium channel and LRP-56 expression at both high and low doses. *In vivo*, the formula improved the serum concentration, reduced the inflow and the release of Ca^2+ ^and inhibited the *LRP *gene expression [118,119].

Four CM formulae, namely *Glycyrrhiza glabra *(GLYC), *Hedyotis diffusa *(OLEN), a formula consisting of 15 herbs including *Cistanche deserticola *(Roucongrong), *Rabdosia rubescens *(Donglingcao) and *Zanthoxylum nitidum *(Liangmianzhen) (SPES), and a formula consisting of eight herbs including *Serenoa repens *(Juyezhong), *Scutellaria baicalensis *(Huangqin), *Panax ginseng *and *Glycyrrhiza glabra *(PC-SPES) were cytotoxic to cancer cell lines in a dose-dependent manner. SPES, PC-SPES, OLEN decreased the *bcl-2 *gene expression and were pro-apoptotic, while GLYC was pro-necrotic without altering the over-expression of bcl-2 in MDR cells. Furthermore, OLEN, SPES and PC-SPES exhibited similar pharmacological effects to etoposide and vincristine [120].

Many MDR reversing alkaloids are also calcium-channel blockers probably because of (1) their structural similarity and (2) inhibition of ABC transporters by the decrease in intracellular calcium concentration. Future research is warranted for potent MDR inhibitors without other pharmacological activities.

Over-expression of ABC transporters and enhanced drug efflux are the causes for MDR. Among the ABC transporters, Pgp is the most extensively studied for its role in MDR reversal effects. While other MDR reversal mechanisms remain unclear, Pgp inhibition is a criterion for further mechanistic study. This article summarises these proposed mechanisms (Additional file 1).

## Conclusion

As some CM active ingredients reverse MDR by directly inhibiting growth and inducing apoptosis in cancer cells, the Pgp-inhibiting CM active ingredients may also be cytotoxic to cancer cells. Future studies should explore not only the MDR reversal effects but also the cytotoxic effects of various CM active ingredients.

## Abbreviations

5-FU: 5-Flurouracil; ABC: Adenosine triphosphate-binding cassette; Ara-C: cytarabine; BCRP/ABCG2: Breast cancer resistance protein; CM: Chinese medicine; DDP: Cisplatin; DNR: Daunorubicin; DOC: Docetaxel; DOX: Doxorubicin; EPI: Epirubicin; FA: Folinic acid; HAR: Harringtonine; HHT: Harringtonolide; LOHP: Oxaliplatin; MDR: Multi-drug resistance; MMC: Mitomycin C; MRP1/ABCC1: Multidrug-resistance-associated protein 1; NFP: Nifedipine; Pgp/ABCB1: P-glycoprotein; PTX: Paclitaxel; TAM: Tamoxifen; VBL: Vinblastine; VCR: Vincristine; VP-16: Etoposide

## Competing interests

The authors declare that they have no competing interests.

## Authors' contributions

SC and GL initiated the review and SC drafted the manuscript. GL and KT revised the manuscript. All authors read and approved the final version of the manuscript.

## Supplementary Material

Additional file 1 **Proposed mechanisms for MDR reversal by the tested Chinese medicinal herbs**. The herbs are grouped into three categories, namely active ingredients, extracts and formulae. Pgp involvement is particularly considered: (+) inhibition on Pgp; (-) no effect on Pgp. Click here for file
