# Supplementary material for: Circumvention of multi-drug resistance of cancer cells by Chinese herbal medicines
Source: Chin Med. 2010 Jul 25;5:26. doi: 10.1186/1749-8546-5-26 (PMC2920269; doi:10.1186/1749-8546-5-26)
Supplement: Additional file 1 — Proposed mechanisms for MDR reversal by the tested Chinese medicinal herbs. The herbs are grouped into three categories, namely active ingredients, extracts and formulae. Pgp involvement is particularly considered: (+) inhibition on Pgp; (-) no effect on Pgp. [file 1749-8546-5-26-S1.DOC]

Additional file 1: Proposed mechanisms for MDR reversal by the CMs tested

| Form studied | Chemical type/ formulation | Name of compound / formulation | Herb | Chemotherapeutic drug with enhanced cytotoxic effect after circumvention of MDR | Proposed mechanism | | Reference |
| --- | --- | --- | --- | --- | --- | --- | --- |
|  |  |  |  |  | Pgp | Others |  |
| Active ingredients in CM | Alkaloid | Tetrandrine | *Stephania tetrandra* | DOX, VCR, DNR, VP-16, Ara-C, VBL | + | Reduce TOPO II expression | 30-35, 37, 38 |
|  |  | Matrine | *Sophora alopecuroides* | DOX, VCR | + | Reduce TOPO II expression, Increase NKG2D-L expression in ABCG2 overexpressed cell | 35, 39, 40 |
|  |  | Tetramethylpyrazine* | *Ligusticium chuangxiong* | DOX, VCR, DNR | +/- |  | 42, 45, 46 |
|  |  | Peimine/Verticine | *Fritillaria thunbergii* | DNR | + |  | 49 |
|  |  | Berbamine | *Mahonia fortunei* | DOX | + | Induce apoptosis,  Increase caspase-3 protein expression, | 50-52 |
|  |  | Dauricine | *Menispermum dauricum* | DOX, VCR | - |  | 95-97 |
|  | Saponin | Total ginsenosides | *Panax ginseng* | DOX, VP-16, Ara-C, HAR | + | No alternation in bcl-2 expression | 55-58 |
|  |  | Total saponins | *Panax notoginseng* | DOX | + |  | 59, 60 |
|  | Flavonoid | Quercetin | *Sophora japonica* | DNR, VBL, PTX | + | Induce apoptosis | 61-64 |
|  |  | Curcumin | *Curcuma longa* | DOX, DNR, VCR, PTX | + | Inhibit Pgp, MRP1 and ABCG2,  Induce apoptosis,  Inhibit FA/BRCA pathway, Decrease bcl-2 and survivin expressions,  Increase caspase-3 expression, Inhibit NFκB pathway | 68-73, 75-77 |
|  |  | Paeonol | *Paeonia suffruticosa* | DOX, DNR, VCR, VBL | - | Induce apoptosis | 100-101 |
|  | Others | Schizandrins | *Schisandra chinensis* | DOX, VCR | + | Increase apoptosis,  Decrease protein kinase C expression | 78-80 |
|  |  | Pseudolaric acid B | *Pseudolarix kaempferi* |  |  | Induce apoptosis,  Disrupt microtubule networks | 104 |
|  |  | Salvinal | *Salvia miltiorrhizae* |  |  | Induce apoptosis,  Inhibit tubulin polymerization | 105 |
| CM extracts |  | Tul-17 |  | VCR | + |  | 85 |
|  |  |  | *Brucea javanica* | DOX, VCR, 5-FU, VP-16, CDDP, MMC | + | Inhibit TOPO II | 86, 87 |
|  |  |  | *Ganoderma species* | DOX, VP-16, DNR |  | Increase intracellular DNA fragmentation,  Induce apoptosis,  Increase caspases 3 and 9 | 115, 116 |
| CM formulae | Injection | Shengmai Injection |  | 5-FU, LOHP, FA, TAM, NEF | + |  | 81, 82 |
|  |  | KLT Injection | *Coix lacryma-jobi* | PTX, DOC | - | Induce apoptosis | 102 |
|  | Powder | Shenghe Powder |  | VCR | + | Induce apoptosis,  Decrease bcl-2 expressions | 83 |
|  |  | Modified Sanwubai Powder |  |  | + | Induce apoptosis,  Decrease p53, bcl-2, rasP21CD44 expressions | 84 |
|  | Others | Sangeng Mixture Decoction |  | DOX | + |  | 88 |
|  |  | R1 |  | DOX | + |  | 90, 91 |
|  |  | Ganai-1 |  | DOX, 5-FU, EPI | + |  | 92 |
|  |  | Tianfoshen |  | DOX | + |  | 93 |
|  |  | Umbilical Plaster |  | 5-FU, MMC, DDP | + |  | 94 |
|  |  | Siwu Mixture |  |  | - |  | 103 |
|  |  | Bushen Huayu Jiedu Formula |  | DDP |  | Prevent inflow and release of Ca^2+^,  Inhibit LRP gene expression | 118, 119 |
|  |  | OLEN, SPES, PC-SPES |  | VCR, VP-16 |  | Induce apoptosis,  Decrease bcl-2 gene expression | 120 |

* Different results obtained by using different cell lines
